# Supplementary material for: Complex‐centric proteome profiling by SEC‐SWATH‐MS
Source: Mol Syst Biol. 2019 Jan 14;15(1):e8438. doi: 10.15252/msb.20188438 (PMC6346213; doi:10.15252/msb.20188438)
Supplement: Supplementary file 7 — Dataset EV6 [file MSB-15-e8438-s007.zip › feature_plots_bioplex/Q9Y2T5.pdf]

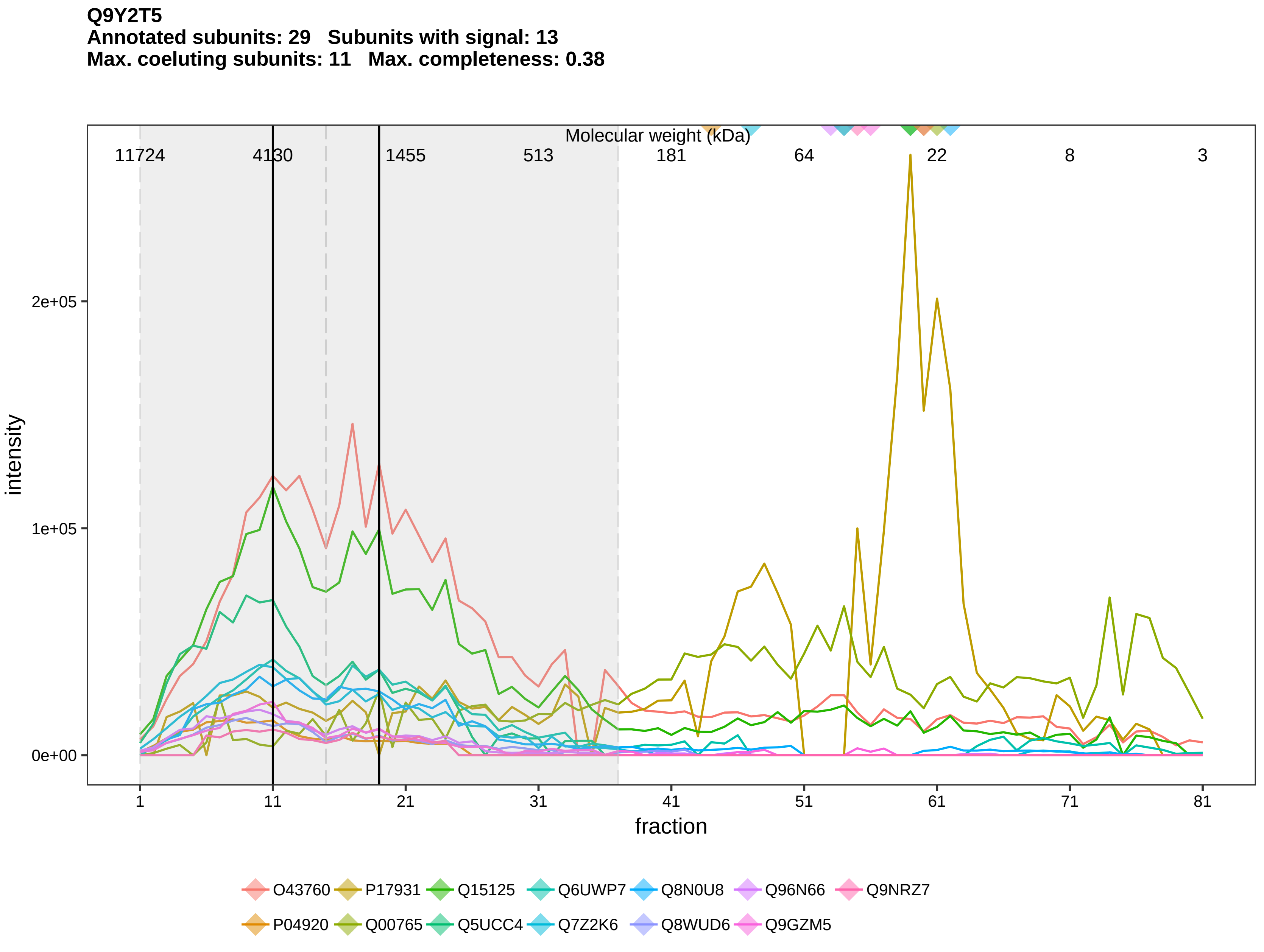

Q9Y2T5
Annotated subunits: 29 Subunits with signal: 13
Max. coeluting subunits: 11 Max. completeness: 0.38
Molecular weight (kDa)
11724
4130
1455
513
181
64
22
8
3
intensity
2e+05
1e+05
0e+00
1
11
21
31
41
51
61
71
81
fraction
O43760
P17931
Q15125
Q6UWP7
Q8N0U8
Q96N66
Q9NRZ7
P04920
Q00765
Q5UCC4
Q7Z2K6
Q8WUD6
Q9GZM5
